# Qualitative Risk Assessment of Foot-and-Mouth Disease Virus Introduction and Transmission to Dairy Farms via Raw Milk Transportation in Thailand: A Scenario-Based Approach

**DOI:** 10.3390/vetsci12070623

**Published:** 2025-06-27

**Authors:** Patidpong Chumsang, Tawatchai Singhla, Warangkhana Chaisowwong

**Affiliations:** 1 Faculty of Veterinary Medicine, Chiang Mai University, Chiang Mai 50100, Thailand; patidpong73@gmail.com (P.C.); tawatchai.singh@cmu.ac.th (T.S.); 2 Participatory One Health Disease Detection (PODD) Centre, Faculty of Veterinary Medicine, Chiang Mai University, Chiang Mai 50100, Thailand; 3 Livestock Strategy and Information Division, Sukhothai Provincial Livestock Office, Department of Livestock Development, Sukhothai 64220, Thailand; 4 Research Center for Veterinary Biosciences and Veterinary Public Health, Faculty of Veterinary Medicine, Chiang Mai University, Chiang Mai 50100, Thailand

**Keywords:** dairy farm, foot-and-mouth disease virus, raw milk transportation, risk assessment

## Abstract

Foot-and-mouth disease (FMD) significantly impacts global livestock industries, with raw milk transportation posing a recognized pathway for viral dissemination, particularly in endemic regions. This study aimed to evaluate the risk of FMD virus (FMDV) introduction and transmission to dairy farms via raw milk transportation in Ban Thi District, Thailand. A qualitative risk assessment methodology, adhering to WOAH guidelines, was employed. Data were collected through structured farmer surveys (*n* = 109), expert interviews (*n* = 12), and reviews of national disease surveillance data and scientific literature. The risk assessment, utilizing a scenario tree approach for domestic dairy cattle, revealed a moderate overall risk of FMDV transmission. This finding is primarily attributed to critical gaps in on-farm biosecurity practices, potential contamination at milk collection centers, and significant challenges in detecting subclinical carrier animals. While the qualitative approach presented inherent limitations and uncertainties, the study successfully highlighted key vulnerabilities. The results underscore the urgent necessity for implementing targeted biosecurity protocols, developing more robust surveillance strategies for FMDV carriers, and establishing standardized risk assessment frameworks to mitigate potential outbreaks and protect the regional dairy industry.

## Simple Summary

Foot-and-mouth disease is a serious threat to livestock worldwide, and raw milk transportation can spread the virus, especially in areas where the disease is common. This study aimed to understand the risk of this disease spreading to dairy farms through raw milk transport in Ban Thi District, Thailand. We used a study approach based on international guidelines, gathering information from farmer surveys (109 participants), expert discussions (12 individuals), and reviewing of government records and scientific papers. Our step-by-step assessment of how the disease could spread in dairy cattle found a moderate overall risk. This risk was mainly due to weaknesses in farm safety practices, possible contamination at milk collection points, and the difficulty in finding animals that carry the virus without showing any signs. Even though our study had some limitations, it clearly showed important areas where the disease could spread. The findings highlight an urgent need for better farm safety rules, improved ways to monitor infected animals, and standard methods to assess such risks, all to prevent future outbreaks and protect local dairy farming.

## 1. Introduction

Foot-and-mouth disease (FMD) is a highly contagious viral infection of cloven-hoofed animals, including cattle, sheep, goats, and pigs, posing a significant global livestock health challenge [1,2]. It is caused by the foot-and-mouth disease virus (FMDV), a member of the Picornaviridae family, known for its rapid spread and severe economic impact on agricultural economies worldwide [3,4]. First reported in Thailand in 1953, the country has since initiated national eradication efforts, facing persistent challenges in achieving disease-free status across all livestock production areas [5,6]. These efforts include the routine application of the Department of Livestock Development (DLD) trivalent vaccines (containing A, O, and Asia 1 serotypes) for cattle, administered three times per year [6]. FMD spreads via multiple routes, such as inhalation of aerosolized virus, direct contact through skin or mucosal abrasions, or ingestion of contaminated feed or water [7,8]. Outbreaks are catastrophic for agriculture, particularly dairy farms, due to high control and eradication costs, significant production losses, and severe trade restrictions on live animals and their products, leading to substantial economic burdens on affected countries and farmers [9,10,11].

In Thailand, FMD is legally defined as an epidemic under the Animal Epidemics Act, B.E. 2558 (2015). This legislation mandates immediate disease control measures upon confirmation of an FMD outbreak, including the imposition of movement restrictions and confinement of infected premises to contain the outbreak and prevent further transmission [12]. Such stringent measures severely impact dairy farmers financially, leading to restrictions on raw milk transportation and additional costs for treatment and biosecurity upgrades [13,14]. A critical concern is the potential for FMDV spread from infected but undetected farms, as FMDV can be present in secretions and excretions of infected animals, including milk, even before clinical signs appear or during the subclinical carrier state [15,16,17]. The role of raw milk transportation in FMDV dissemination, via fomites (e.g., contaminated vehicles, equipment) and mechanical transmission by personnel, has been frequently implicated in outbreaks in various countries, highlighting its significance as a high-risk pathway [18,19,20]. Despite the application of movement restrictions, new outbreaks can still occur [21,22,23], suggesting that banning movement alone may be insufficient for comprehensive disease mitigation across the entire dairy farming industry. The precise risk of FMD transmission via raw milk transportation, particularly in endemic settings such as Thailand where FMDV circulates continuously, remains an area requiring clearer understanding and targeted assessment [24,25].

Risk analysis, a formal and systematic process for evaluating the risks associated with the introduction or spread of infectious agents in an animal health context [26,27], is crucial for informed decision-making in disease control. Risk assessment, a key component of risk analysis, can be applied even with limited data, though its effectiveness relies heavily on the selection of an appropriate risk model, which may involve assumptions and subjective choices [28,29]. This approach is widely used in veterinary epidemiology to assess risks in animal and animal product importation or to evaluate the transmission dynamics of infectious diseases within populations [30,31]. The outcomes of risk estimation provide scientific evidence to inform the development of proactive disease control measures, particularly when solid empirical evidence is insufficient, by incorporating expert opinions from multiple sources [32,33].

FMD continues to pose a persistent challenge for dairy farmers in endemic regions, including Ban Thi District, Lamphun Province, Thailand. Raw milk transportation, an essential routine practice integral to dairy farm operations and the broader dairy value chain, represents a potential and often overlooked pathway for FMDV transmission [34,35]. Existing control measures have not entirely prevented recurrent outbreaks in this area, underscoring the urgent need for a comprehensive evaluation of specific risk factors associated with this critical pathway. Understanding these localized risks is paramount for developing targeted and effective strategies to prevent disease spread, mitigate significant economic losses, and enhance the robustness of national FMD control legislation and biosecurity frameworks. Therefore, this study aimed to assess the risk of FMDV introduction and transmission through raw milk transportation, leveraging current data and assumptions to provide scientific evidence for improving biosecurity practices and informing policy frameworks in Thailand. The findings are anticipated to demonstrate the risk of FMDV transmission via this pathway, thereby underscoring the necessity for enhanced biosecurity, improved surveillance for carrier animals, and standardized risk assessment frameworks.

## 2. Materials and Methods

### 2.1. Study Area

**Figure 1:**
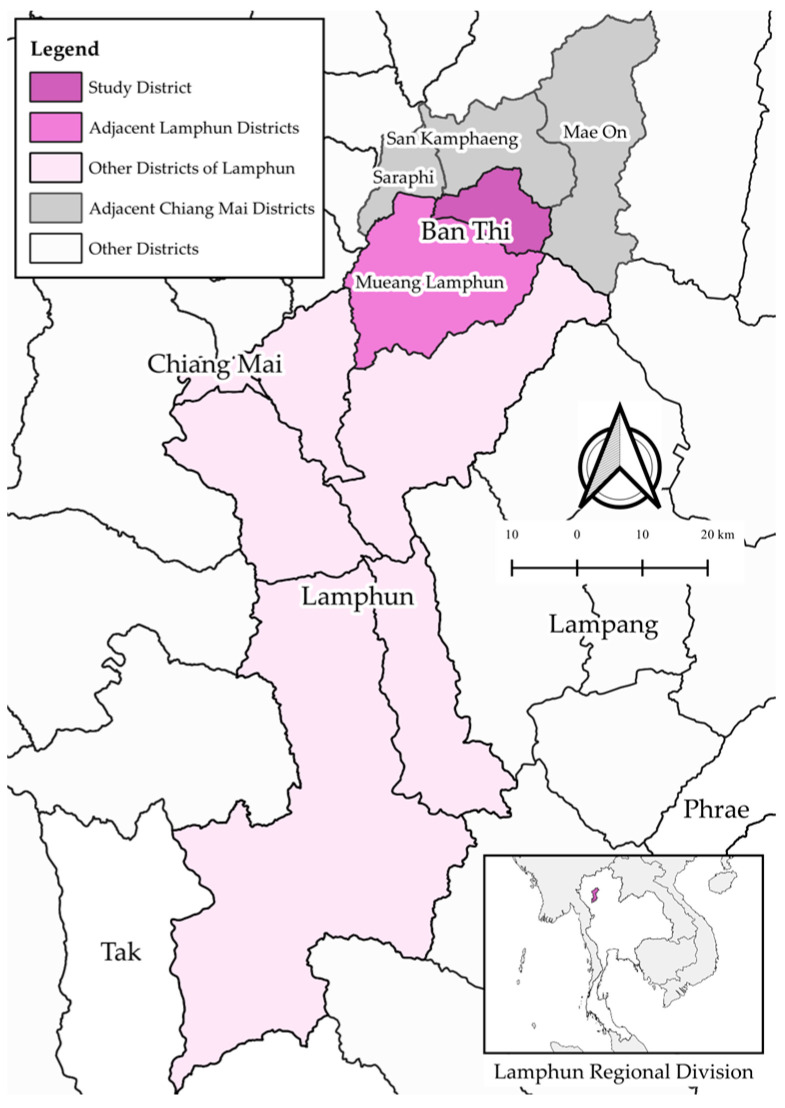
Map of the study area, Ban Thi District, Lamphun Province, Thailand. The map illustrates the geographical location of Ban Thi (study district) relative to surrounding districts in Lamphun Province (Mueang Lamphun, other districts of Lamphun) and key adjacent districts in Chiang Mai Province (San Kamphaeng, Mae On, Saraphi). It also indicates the broader regional context within Thailand (inset map). The map was generated using QGIS.

The study area is located in Ban Thi District, Lamphun Province, Thailand (Figure 1). This region is notable for its dairy production and is classified as FMD-endemic [36] making it representative of FMD-endemic areas in Thailand. Ban Thi District covers an area of approximately 122.45 square kilometers and shares borders with three important districts in Chiang Mai Province: San Kamphaeng, Mae On, and Saraphi Districts. It is also well-connected by the Chiang Mai–Lampang superhighway. The selection of Ban Thi District as the study area was based on its history of consistently reported FMD outbreaks and high FMD prevalence [36]. These factors make Ban Thi District a relevant and important location for studying the risk of FMD transmission in the Thai dairy industry.

### 2.2. Scope of Study and Study Design

This study investigated the potential introduction of foot-and-mouth disease virus (FMDV) to dairy farms in Ban Thi District, Lamphun Province, Thailand, via raw milk transportation. A cross-sectional study was conducted between May and June 2024, employing a qualitative risk assessment framework alongside survey data collected from local dairy farmers. The risk assessment evaluated FMDV contamination pathways, from infected cattle, through raw milk transportation (specifically the movement of milk from infected farms to milk collection centers), to dairy farms, and the potential impact of outbreaks on dairy cattle.

The study population comprised members of dairy cooperatives in the district. Eligible participants were dairy farmers, aged over 18 years, with farms located in Ban Thi District, and registered members of a local dairy cooperative (limited or private company). Prior approval was obtained from relevant agencies. All participants were informed of the study objectives and assured of confidentiality. Oral consent was obtained before administering a structured questionnaire.

Expert opinions were collected using a structured questionnaire. Experts were selected based on their experience with FMD and were drawn from various institutions, including universities; the Department of Livestock Development (specifically, the Animal Health Division of the Fifth Regional Livestock Office, the Livestock Office of Chiang Mai, Sukhothai, and Ban Thi District); the Veterinary Research and Development Center (Upper Northern Region); the Lamphun Animal Quarantine Station; and local milk collection centers. The questionnaire was designed to gather information on FMDV, local dairy management practices, and potential risk factors for disease spread via routine raw milk transportation processes in Ban Thi District, Lamphun Province, Thailand.

### 2.3. Risk Assessment Framework and Risk Questions

The study mainly followed the framework set by the WOAH handbook on import risk analysis [33]. The framework comprises three key components including, entry assessment, exposure assessment, and consequence assessment. Likelihood estimates for each step within the risk pathways were derived from literature, observation, and expert opinion. Additionally, expert opinion was consulted to provide supplementary data and information necessary to comprehensively address the risk question. The overall questions of the risk assessment were defined as: What is the likelihood of the FMDV from an infected but undetected dairy farm being introduced into the milk collection center via routine raw milk transportation? In case FMDV was introduced into the milk collection center, what would be the likelihood of onward transmission of the FMDV into the other dairy farms in Ban Thi District of Lamphun Province? The susceptible species considered in this risk assessment is domestic dairy cattle.

### 2.4. Hazard Identification

FMDV exhibits a pattern of endemicity in Ban Thi District, Lamphun Province, Thailand, with documented annual outbreaks [36]. This finding supports the identification of FMDV as a potential hazard in this scenario. Additionally, FMD is listed as an epidemic disease under the Animal Epidemic Act of Thailand (B.E. 2558; 2015), necessitating quarantine and movement restrictions for carcasses to prevent disease spread and economic losses to dairy farmers. Furthermore, data obtained from milk collection center officers in the study area indicate that dairy farms in Ban Thi District can potentially produce and transport approximately 41 tons of raw milk daily. Since raw milk has been identified as a potential vehicle for FMDV transmission [15], its presence in the context of FMD endemicity strengthens the case for FMDV in raw milk as a hazard.

### 2.5. Physical Risk Pathway and Scenario Trees

**Figure 2:**
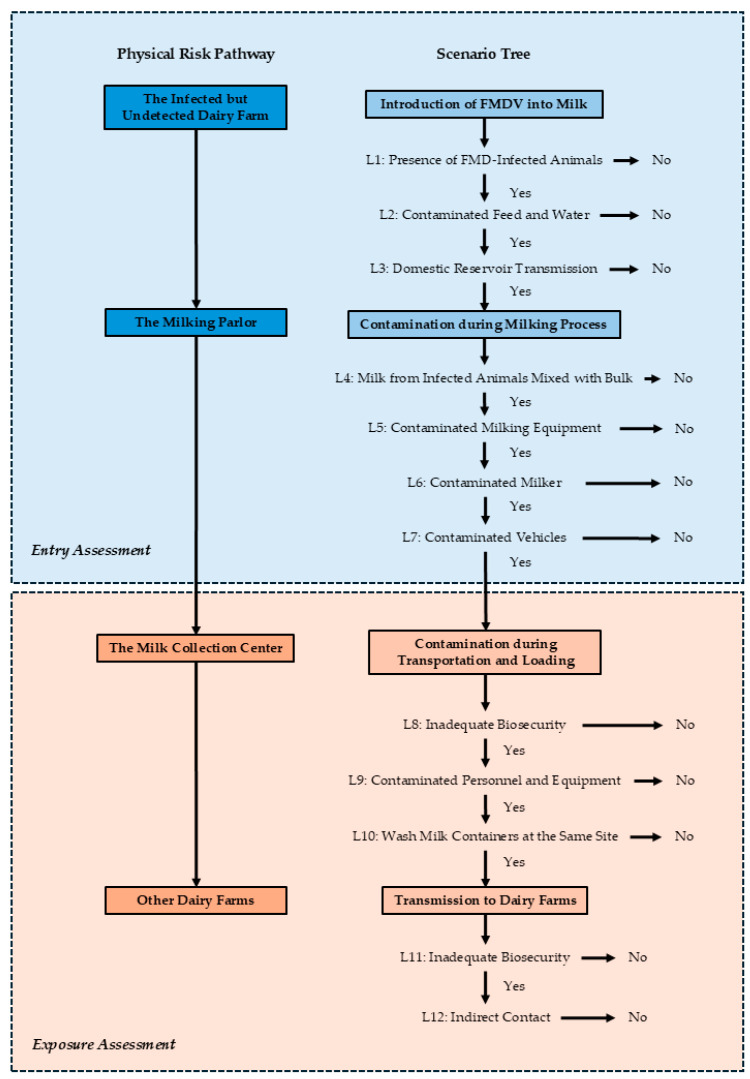
Physical risk pathway and scenario tree for the introduction and transmission of foot-and-mouth disease virus (FMDV) to dairy farms via raw milk transportation in Ban Thi District, Lamphun Province, Thailand. The diagram illustrates the sequential events of FMDV entry and exposure, categorized into entry assessment (from an infected farm to the milk collection center) and exposure assessment (from the milk collection center to another dairy farm). The scenario tree details specific likelihoods (L1–L12) at each critical juncture of the pathway.

Figure 2 depicts the potential pathway for the movement of FMDV originating from an infected but undetected dairy farm to other farms within Ban Thi District. This comprehensive scenario tree was developed based on current practices employed by local dairy farmers. The diagram is structured into two main components which includes a physical risk pathway, illustrating the sequential flow of raw milk transportation, and a corresponding scenario tree, detailing the potential events and associated likelihoods of FMDV transmission at each stage. The scenario tree incorporates the potential contamination of raw milk from infected cattle during collection as an initial entry point for the virus. This section, along with subsequent events leading to contamination during milking, constitutes the entry assessment. The subsequent transportation stage of raw milk to the collection center is identified as a potential exposure route for other farms, encompassing contamination during transportation and loading, and subsequent transmission to other dairy farms. This part of the scenario is categorized within the exposure assessment. The scenario tree provides a robust framework for assessing the movement of the hazard (FMDV) within the system and determining the likelihood of the virus traversing each parameterization (L1–L12).

### 2.6. Data Collection and Parameterizations

Data collection for this qualitative risk assessment employed a triangulation approach to enhance reliability. Primary data regarding raw milk transportation practices were obtained directly from 109 dairy farmers in Ban Thi District using a structured questionnaire developed based on previous work [37]. The questionnaire was pre-tested on a random sample of ten respondents from the neighboring Mae Wang District, Chiang Mai Province, to ensure clarity and comprehensibility. Secondary data on FMD control measures, national statistics, and relevant legislation were sourced from government databases, documentation, and regulatory programs. A comprehensive search of published literature was conducted from November 2024 to April 2025, utilizing databases such as PubMed, Scopus, and ScienceDirect, which served as a primary source for validating the information gathered from these documents. To address information gaps, expert opinion was sought from twelve qualified individuals with experience in FMD from various sources. Interviews were conducted to collect expert opinions on specific aspects of the risk assessment for each parameterization. Data from all sources (farmer questionnaires, secondary data, and expert opinions) were entered into and coded for confidentiality in Microsoft Excel spreadsheets. After entry, data were checked for errors before being matched to the corresponding parameterizations within the risk pathway framework for further analysis. The data and evidence used to derive each likelihood are presented in the parameterizations below.

#### 2.6.1. Entry Assessment

The likelihood of entry of FMDV to other susceptible dairy cattle from infected but undetected dairy farms was assessed by considering the likelihood of the presence of FMD-infected animals; the likelihood of contaminated feed and water; the likelihood of domestic reservoir transmission; the likelihood of milk from infected animals mixed with bulk; the likelihood of contaminated milking equipment; the likelihood of a contaminated milker during the milking process; and the likelihood of a contaminated vehicle during the milking process.

- Parameterization of L1: Likelihood of presence of FMD-infected animals. This parameter assessed the presence of FMD-infected animals in the district, based on FMD prevalence data, animal management practices from farmer questionnaires (*n* = 109), and expert input.
- Parameterization of L2: Likelihood of contaminated feed and water. This parameter evaluated the likelihood of contaminated feed and water, considering roughage sourcing, transportation, storage, water sources, and disinfection practices, identified through farmer surveys.
- Parameterization of L3: Likelihood of domestic reservoir transmission. This parameter assessed domestic reservoir transmission, considering FMD prevalence in other livestock, farm location near high-risk areas, and biosecurity measures against various domestic animals and pests.
- Parameterization of L4: Likelihood of milk from infected animals mixed with bulk. This parameter determined the likelihood of milk from infected animals contaminating bulk milk, based on farmer awareness of FMD clinical signs (including their ability to identify key signs), daily animal observation, understanding of subclinical FMD carrier animals, and mitigation practices such as isolation and separate milking.
- Parameterization of L5: Likelihood of contaminated milking equipment. This parameter assessed the likelihood of contaminated milking equipment, considering practices such as milking order for infected animals, use of separate equipment, shared pens, and equipment cleaning procedures.
- Parameterization of L6: Likelihood of contaminated milk during the milking process. This parameter evaluated milker contamination during milking based on personal protective equipment (PPE) usage (gloves, hairnets, masks, aprons), farm staffing (owners/workers as milkers/transporters), and post-milking hygiene practices.
- Parameterization of L7: Likelihood of contaminated vehicles during the milking process. This parameter assesses contaminated vehicles during milking, considering vehicle types, proximity to milking pens, use of disinfection points, and regular vehicle cleaning practices.

#### 2.6.2. Exposure Assessment

Parameters examined to determine the likelihood of exposure of FMDV into susceptible dairy farms via raw milk transportation were the likelihood of inadequate biosecurity at milk collection centers, the likelihood of contaminated personnel and equipment, and the likelihood of washing containers at the same site, while for transmission to dairy farms, there were the likelihood of inadequate biosecurity at dairy farms and the likelihood of indirect contact with dairy cattle.

- Parameterization of L8: Likelihood of inadequate biosecurity at milk collection center. This parameter evaluated inadequate biosecurity at milk collection centers by assessing risks from milk delivery personnel, monitoring wheel-dipping ponds, and systematically inspecting equipment brought into the center.
- Parameterization of L9: Likelihood of contaminated personnel and equipment. This parameter assessed contaminated personnel and equipment at the milk collection center, focusing on virus transmission risks during raw milk receiving, staff PPE usage, and shared equipment acting as fomites.
- Parameterization of L10: Likelihood of washing milk containers at the same site. This parameter evaluated the likelihood of milk container washing occurring at the milk collection center by comparing on-farm washing practices to regulations, considering disease transmission risks, and noting pandemic-related changes in delivery protocols.
- Parameterization of L11: Likelihood of inadequate biosecurity at dairy farms. This parameter assessed inadequate biosecurity at dairy farms by evaluating risks from individuals entering the farm (e.g., milk drivers from various exposure settings), monitoring of wheel-dipping ponds, and personal hygiene practices of farm personnel.
- Parameterization of L12: Likelihood of indirect contact with dairy cattle. This parameter evaluated the likelihood of indirect contact with dairy cattle, considering farmer engagement in close-contact farm activities upon return from milk collection and the proximity of on-farm milk container washing to animal pens.

#### 2.6.3. Consequence Assessment

The consequence assessment evaluated the potential impact of an FMDV outbreak on dairy farms in Ban Thi District. This evaluation included a consideration of both direct and indirect economic losses. The assessment drew upon data regarding the district’s dairy industry, including the number of raw milk collection centers, daily raw milk production, the number of dairy farms, and the population of dairy cows [38,39]. Information on the economic and cultural value of livestock products in Lamphun Province was also included [38]. The Animal Epidemics Act, B.E. 2558 (2015), was reviewed to understand the regulations regarding farm closures and cessation of milk deliveries during disease outbreaks. Previous studies on economic losses from FMD in the district were analyzed to quantify potential unsold milk values, decreased raw milk purchase prices, and additional costs incurred by dairy farmers during outbreaks [13].

### 2.7. Combination of the Likelihood of the Occurrence Hazard

The risk assessment employed a scenario tree approach to depict the potential events along the raw milk transportation pathway. Each event within the scenario tree was characterized by a set of parameters. The likelihood of occurrence for each event was categorized based on a descriptive scale including negligible (rare and can be ignored), low (possible but uncommon), medium (regular occurrence), and high (very frequent occurrence) [40]. Event likelihoods were then combined with other parameters using a risk matrix (Table 1) [32]. Also, the results of the likelihood of the occurrence of hazard were then combined using a risk matrix (Table 1).

### 2.8. Combination of Risk Estimation

Finally, the likelihood of the occurrence hazard and the consequence level were then combined using a risk matrix (Table 2) to determine the overall risk of FMDV introduction to dairy farms via raw milk transportation [41].

### 2.9. Uncertainty Assessment

Following the development of the risk pathway and scenario trees, the likelihood of occurrence and the associated uncertainty of the supporting evidence were assessed. Uncertainty levels were assigned to each node based on the definitions outlined in Table 3 [29]. This step aims to mitigate potential misinterpretations and overconfidence in the risk assessment.

## 3. Results

### 3.1. Introduction of FMDV into Milk

#### 3.1.1. Likelihood of Presence of FMD-Infected Animals (L1)

Assessed as low with a low level of uncertainty, the presence of FMD-infected animals in the district considered varying FMD prevalence rates of 18.22% between 2003 and 2004 [24], 35.89% in 2015, and 39.34% in 2016, respectively [25], as well as documented outbreaks in Ban Thi and neighboring districts [36]. While farmers reported high rates of animal source checks at 76.15% (83/109), quarantine of new animals at 61.47% (67/109), vaccination at 84.40% (92/109), and daily health checks at 94.50% (103/109), a critical gap was identified in the absence of measures to detect FMD carrier animals.

#### 3.1.2. Likelihood of Contaminated Feed and Water (L2)

A low likelihood with moderate uncertainty was assessed for contaminated animal feed and water. This was based on findings that a majority of farmers sourced roughage both internally and externally (77.98%, 85/109), with (84.40%, 92/109) cultivating feed on-farm. Concerns included the transportation of roughage without covering truck beds. While most farms used disinfectant sprays or wheel-dipping ponds (90%) and had dedicated feed storage rooms, some lacked dedicated feed carts. Groundwater was widely used (89.91%, 98/109), and a considerable proportion of farms (76.15%, 83/109) reported a history of disease outbreaks.

#### 3.1.3. Likelihood of Domestic Reservoir Transmission (L3)

Domestic reservoir transmission was assessed as having a low likelihood with a low level of uncertainty. This considered historical pig FMD prevalence to be 11.11% during 2003–2004 [24] and reported FMD outbreaks in beef cattle and buffalo in neighboring districts [36]. Many farms were located in high-risk areas adjacent to agricultural zones utilizing manure or near livestock markets. Despite most farms having a single entrance/exit, inadequate biosecurity measures concerning reservoir control were observed, with most lacking comprehensive plans for rodents, birds, and insects. Poor farm sanitation and the common presence of domestic animals were also noted.

### 3.2. Contamination During Milking Process

#### 3.2.1. Likelihood of Milk from Infected Animals Mixed with Bulk (L4)

Milk from infected animals contaminating bulk milk was assessed as having a high likelihood with a high level of uncertainty. While farmers demonstrated high awareness of FMD clinical signs, with 92.66% (101/109) identifying key signs and 94.50% (103/109) performing daily animal observations, a significant knowledge gap existed regarding subclinical FMD carrier animals, with ~90% (98/109) having limited understanding. Mitigation practices included isolating suspected cases 82.57% (90/109), milking infected cows at the last, 88.99% (97/109), and using dedicated equipment for infected cows, 61.47% (67/109). Most farmers reported never selling raw milk from clinically infected animals, 93.58% (102/109), and ceasing deliveries upon detection, 83.49% (91/109).

#### 3.2.2. Likelihood of Contaminated Milking Equipment During Milking Process (L5)

Contaminated milk collection equipment was assessed as having a low likelihood with a high level of uncertainty. This was based on 88.99% (97/109) of farmers milking infected animals at the last order and 61.47% (67/109) using separate milking equipment. However, shared milking and keeping pens were common. Cleaning practices varied, with 57.80% (63/109) of farmers dipping the rubber liner in clean water and 89.91% (98/109) lifting milk containers without cleaning their external surfaces.

#### 3.2.3. Likelihood of Contaminated Milker During Milking Process (L6)

Milker contamination during milk collection was assessed as moderate with a high level of uncertainty. This was primarily due to low consistent usage of PPE (e.g., gloves 26.61% (29/109), hairnets 42.20% (46/109), masks 26.61% (29/109), and aprons 34.86% (38/109)). Furthermore, a significant proportion of farmers, 70.64% (77/109), reported not changing clothes before leaving the farm, and 49.54% (54/109) did not change their shoes.

#### 3.2.4. Likelihood of Contaminated Vehicles During Milking Process (L7)

Vehicle contamination during milk collection was assessed as low with a high level of uncertainty. Motorcycles with sidecars were common, with 75.23% (82/109) of farmers bringing them directly into or near milking pens. Consistent use of wheel-dipping ponds was not observed. Furthermore, 49.54% (54/109) of farmers reported not regularly cleaning their vehicles, and none used disinfectants for vehicle interiors. These findings, along with the understanding that vehicles and equipment can act as fomites for FMDV transmission, led to this assessment.

### 3.3. Contamination During Transportation and Loading

#### 3.3.1. Likelihood of Inadequate Biosecurity at Milk Collection Center (L8)

Inadequate biosecurity at the milk collection center was considered low, with a moderate level of uncertainty. This assessment highlighted potential FMDV exposure risks from milk delivery personnel who may have been exposed on farms or in nearby contaminated areas (e.g., agricultural areas using animal manure and livestock markets). While wheel-dipping ponds with disinfectants were present, the duration of vehicle contact with the disinfectant solution was rarely monitored. Inadequate biosecurity measures also included a lack of systematic inspections for equipment brought in by dairy farmers, such as mobile phones, milking equipment, and documents.

#### 3.3.2. Likelihood of Contaminated Personnel and Equipment (L9)

Contaminated personnel and equipment at the milk collection center were considered moderate with a high level of uncertainty. This was attributed to virus transmission risks during raw milk receiving due to interactions between staff and delivery personnel, inadequate PPE usage (e.g., infrequent use of gloves and aprons), and shared equipment (sledgehammers and dippers) acting as fomites.

#### 3.3.3. Likelihood of Washing Milk Containers at the Same Site (L10)

Milk container washing occurring at the milk collection center was assessed as low, with a high level of uncertainty. This was primarily due to 65.14% (71/109) of farmers washing containers on-farm, suggesting a discrepancy with regulatory requirements for on-site washing at collection centers. While on-farm cleaning may improve individual farm hygiene, potential disease transmission risks from contaminated containers or residual milk exist. However, 77.98% (85/109) of farmers returned directly to their farms after milk delivery, potentially reducing cross-contamination at the center. The prevalence of on-farm washing increased around 2020, coinciding with COVID-19 pandemic protocols implemented to minimize contact between farms.

### 3.4. Transmission to Dairy Farms

#### 3.4.1. Likelihood of Inadequate Biosecurity at Dairy Farms (L11)

Inadequate biosecurity at dairy farms was considered low with a high level of uncertainty. Key risk factors included the potential for virus introduction by individuals entering the farm, specifically milk containers delivered directly by drivers who may have been exposed in various settings (milk collection centers, other farms, agricultural areas, and livestock markets). While wheel-dipping ponds were used at farm entrances, vehicle contact duration with disinfectants was often not monitored. Inadequate personal hygiene among farm personnel (many not changing clothes or shoes) and the absence of disinfection measures for potential fomites such as mobile phones and wallets were also observed.

#### 3.4.2. Likelihood of Indirect Contact with Dairy Cattle (L12)

Indirect contact with dairy cattle was considered high with a high level of uncertainty. This was based on 88.07% (96/109) of farmers engaging in close-contact farm activities (pen cleaning, feeding, and vaccination) upon their return from the raw milk collection center. Furthermore, 65.14% (71/109) of farmers washed their milk containers on the farm, often in close proximity to animal pens.

The qualitative risk assessment evaluated the likelihood of FMDV introduction and transmission via raw milk transportation in Ban Thi District. The key findings for each parameterization (L1–L12), including relevant percentages and observations, along with their assessed likelihoods and uncertainty levels, are summarized in Table 4.

### 3.5. Consequence Assessment

The consequence assessment revealed that an FMDV outbreak in Ban Thi District has the potential to cause significant economic and social impacts, with economic impacts being more substantial [10]. Direct losses, such as a reduction in milk production (potentially reaching upwards of 80% in chronically infected cattle), would severely affect the district’s dairy industry [42]. The Animal Epidemics Act’s mandate to stop moving animals and sending raw milk from infected farms would lead to substantial revenue losses for farmers (Animal Epidemics Act, B.E. 2558 (2015)). Previous studies indicated significant unsold milk values and decreased raw milk purchase prices during outbreaks [13]. Indirect losses would include increased costs for activities such as treatment, vaccination, labor, disinfection, and antibiotic residue testing [13]. Furthermore, outbreaks would lead to foregone revenue due to restrictions on market access [13,43]. The consequence of the introduction and transmission of FMDV to dairy farms via raw milk transportation in Ban Thi District was rated as high with a low level of uncertainty.

### 3.6. Risk Estimation

The likelihood of FMDV introduction to susceptible dairy cattle in Ban Thi District through raw milk transportation from an undetected but infected dairy farm was estimated by combining the likelihood levels of all nodes within the entry and exposure assessment pathways. This combined estimate represents the likelihood of a hazard occurring. The overall risk estimation for FMDV introduction and transmission via raw milk transportation was derived by combining the likelihood of the hazard’s occurrence with the likelihood of its associated consequences.

#### 3.6.1. Likelihood of the Occurrence of Hazard

This analysis evaluates the combined conditional events of FMDV entry into the milk collection center and subsequent exposure of susceptible dairy cattle on other farms via raw milk transportation. The entry assessment, originating from an undetected infected dairy farm, is determined by the likelihood of FMDV introduction into milk (low with low uncertainty) and contamination during milk collection (low with high uncertainty). This results in an overall entry likelihood rated as low with moderate uncertainty. The exposure assessment, from the milk collection center to susceptible dairy cattle on other farms, is influenced by the likelihood of contamination during transportation and loading (low with moderate uncertainty) and the likelihood of transmission to dairy farms (low with high uncertainty). This leads to an overall exposure likelihood rated as low with moderate uncertainty. Consequently, the overall hazard occurrence likelihood, encompassing both entry and exposure, is rated low with moderate uncertainty (Table 5).

#### 3.6.2. Risk Estimation for Introduction and Transmission of FMDV to Dairy Farms via Raw Milk Transportation

The overall risk assessment combines the likelihood of hazard occurrence (low with moderate uncertainty) with the assessed consequences of an outbreak (high with low uncertainty). Utilizing a combination matrix as prescribed in Table 2 [41], the overall risk is rated moderate with a moderate level of uncertainty (Table 6).

## 4. Discussion

### 4.1. Overall Risk Assessment and Key Vulnerabilities

FMD significantly burdens global livestock economies due to direct and indirect losses [10,44,45]. Raw milk transport is a critical, often undetected, FMDV pathway, fostering viral spread and complicating tracing [12,46,47,48]. Effective prevention demands robust biosecurity, active surveillance, rapid response, and tailored risk communication across the raw milk value chain to enhance sustainability and livelihoods [35,49,50,51,52,53].

This study, adhering to WOAH guidelines [33], revealed a moderate overall FMD risk with moderate uncertainty via raw milk transport in Ban Thi District. It is important to note that many individual likelihood parameters (Ls) within this assessment were characterized by a high level of uncertainty, reflecting data limitations and the inherent complexities of the system. This finding necessitates the evaluation and implementation of mitigation strategies prior to authorizing raw milk transportation within the district [41], while also underscoring the critical need for further research to reduce these uncertainties before a definitive conclusion on the precise level of risk can be established. Key vulnerabilities—insufficient FMD carrier testing, farmer over-reliance on clinical signs, and virus transmission via contaminated milk—underscore the urgent need for systematic surveillance and consistent biosecurity.

### 4.2. Analysis of Likelihoods and Contributing Factors

FMDV transmission was consistently ‘low likelihood’ at several nodes due to existing mitigation, although uncertainty varied. For L1 (presence of FMD-infected animals), low likelihood (low uncertainty) reflects high farmer adherence to animal source checks (76.15%), quarantine (61.47%), vaccination (84.40%), and daily health monitoring (94.50%), which reduces immediate introduction risk [54,55,56]. L2 (contaminated feed and water) showed low likelihood (moderate uncertainty), supported by prevalent on-farm feed cultivation (84.40%) and disinfection protocols (90% sprays, 89.91% groundwater use), despite concerns over uncovered external roughage transport (77.98% external sourcing) and historical disease presence (76.15% farms with disease history) [51,57,58,59]. L3 (domestic reservoir transmission) also presented low likelihood (low uncertainty), based on historically low pig FMD prevalence (11.11% in 2003–2004) [6] and most farms using single entry/exit points, which limit external contact, despite some adjacent high-risk areas and a lack of comprehensive pest plans [60,61,62,63,64].

In the milking process, L5 (contaminated milking equipment) showed low likelihood (high uncertainty). Supported by most farmers milking infected animals last (88.99%) and using separate equipment (61.47%), which reduces direct transmission, L5 showed low likelihood (high uncertainty). However, shared pens, inconsistent rubber liner dipping (57.80%), and uncleaned external container surfaces (89.91%) introduce high uncertainty, given FMDV persistence on surfaces [65] and the need for proper disinfection [15,66], reflecting general biosecurity inconsistencies [67]. L7 (contaminated vehicles during the milking process) was low likelihood (moderate uncertainty). While vehicles are generally kept near milking areas with some disinfection [68], inconsistent wheel-dipping and infrequent vehicle cleaning (49.54% not regular) without interior disinfection create uncertainty, as vehicles are known fomites [67,69].

At the milk collection center, L8 (inadequate biosecurity) had a low likelihood (moderate uncertainty) due to existing measures such as wheel-dipping ponds and its primary role as a reception point, aligning with the benefits of biosecurity intervention [70]. However, inconsistent monitoring and equipment inspection lead to uncertainty, reflecting “imperfect compliance” [71]. L10 (washing milk containers at the same site) had a low likelihood (high uncertainty) as most farmers wash containers on-farm (65.14%), particularly since COVID-19 protocols (77.98% return directly), reducing centralized contamination risk [15,72]. This shifts risk to on-farm practices if cleaning is inadequate. L11 (inadequate biosecurity at dairy farms) had a low likelihood (high uncertainty), supported by general farmer awareness and some wheel-dipping pond use [70]. Yet, high uncertainty arises from significant inconsistencies including inadequate personal hygiene (e.g., farm personnel not changing clothes or shoes) and no disinfection for personal fomites, demonstrating critical behavioral and practical challenges in adherence [71].

Conversely, certain nodes exhibited higher likelihoods or significant uncertainties, pinpointing key vulnerabilities. L4 (milk from infected animals mixed with bulk) had a high likelihood (high uncertainty). Despite high farmer awareness of clinical signs (92.66%) and daily observation (94.50%), a critical ~90% knowledge gap on subclinical FMD carriers existed. This is crucial as FMDV sheds in milk from subclinical or carrier animals before or without overt clinical signs [15,73]. Infected milk is a potent source of infection, and even low viral levels can cause widespread contamination when pooled [20,74,75,76]. Undetected carriers, due to reliance on clinical signs and the absence of routine testing, perpetuate viral circulation, contaminate milk, and induce economic losses, posing a major challenge in endemic FMD control [3,4,16,77,78]. Thus, rigorous serological/virological testing and routine milk screening are crucial [78,79].

L6 (contaminated milker during milking process) had a moderate likelihood (high uncertainty), primarily due to low consistent PPE usage (gloves 26.61%, hairnets 42.20%, masks 26.61%, aprons 34.86%) and inadequate personal hygiene (70.64% not changing clothes, 49.54% not changing shoes before leaving farm) [80,81]. Personnel act as mechanical vectors [82,83,84], a challenge also noted in cattle transport drivers [69]. L9 (contaminated personnel and equipment at the milk collection center) also had a moderate likelihood (high uncertainty), stemming from staff-delivery personnel interactions, inadequate PPE, and shared equipment acting as fomites [85,86,87]. FMDV can persist on various surfaces, making shared equipment a significant cross-contamination risk [88,89].

Finally, L12 (indirect contact with dairy cattle) had a high likelihood (high uncertainty), based on 88.07% of farmers engaging in close-contact activities (e.g., pen cleaning) upon returning from milk collection and 65.14% washing milk containers near animal pens [45,90]. These practices increase FMDV transmission risk from contaminated clothing, footwear, or equipment to susceptible animals [82,83,84]. These findings underscore the urgent need for targeted interventions focusing on subclinical carrier detection, consistent PPE use, and strict hygiene protocols to reduce overall FMDV transmission risk in the raw milk value chain.

### 4.3. Limitations and Future Research

This qualitative risk assessment for FMDV transmission via raw milk transportation in Ban Thi District, while insightful, has limitations. Subjectivity can influence risk categorization and likelihood interpretation [26,40], and the lack of standardized frameworks coupled with challenges in combining qualitative likelihoods can introduce uncertainty [28,29]. Our two-stage combination matrix, while providing granular assessment aligned with scenario tree methodologies [31,32,33,41,43], may yield a lower overall likelihood than single-step methods, as sequential ‘low’ or ‘moderate’ likelihoods can cumulatively reduce the final estimate. Limited published FMD risk analyses in Thailand constrained contextual comparisons.

Despite these limitations, our qualitative approach successfully integrated expert opinions and site-specific observations, providing a nuanced understanding of local practices and transmission pathways. Future studies should incorporate quantitative data (e.g., epidemiological surveillance, milk testing) to enhance precision. Developing standardized FMD transmission risk assessment frameworks for Thailand and establishing a national database would facilitate comparative analysis and collaborative data sharing, fostering a more comprehensive understanding of FMD risk factors and transmission pathways.

## 5. Conclusions

This study assessed the risk of FMDV transmission via raw milk transportation in Ban Thi District, Thailand, utilizing a qualitative risk assessment approach. The findings indicate a moderate risk of FMDV transmission, primarily driven by critical gaps in on-farm biosecurity practices, the potential for contamination at milk collection centers, and the significant challenges associated with detecting subclinical carrier animals. While the qualitative methodology presented inherent limitations and uncertainties, the study successfully highlighted key vulnerabilities within the raw milk transportation pathway. This underscores the urgent necessity of implementing targeted and enhanced biosecurity protocols, developing more robust surveillance strategies for carrier animals, and establishing standardized risk assessment frameworks. This research provides valuable insights into the complex dynamics of FMDV spread through raw milk transportation, emphasizing that sustained efforts in prevention and control are essential to mitigate potential outbreaks and protect the dairy industry and associated livelihoods in the region.

## Figures and Tables

**Table 1:** Combination matrix used for descriptive likelihoods [32].

**Results of the Assessment of Parameter 1**	**Results of the Assessment of Parameter 2**			
	Negligible	Low	Moderate	High
Negligible	Negligible	Negligible	Negligible	Negligible
Low	Negligible	Low	Low	Low
Moderate	Negligible	Low	Moderate	Moderate
High	Negligible	Low	Moderate	High

**Table 2:** Combination matrix for risk estimation [41].

**Results of the Assessment of Parameter 1**	**Results of the Assessment of Parameter 2**			
	Negligible	Low	Moderate	High
Negligible	Negligible	Low	Low	Moderate
Low	Low	Low	Moderate	Moderate
Moderate	Low	Moderate	Moderate	High
High	Moderate	Moderate	High	High

**Table 3:** Uncertainty categories for parameter value estimates depending on data availability [29].

Uncertainty Category	Interpretation
Low	There are solid and complete data available; strong evidence is provided in multiple references; authors report similar conclusions. Several experts have multiple experiences of the event, and there is a high level of agreement between experts.
Moderate	There are some but not complete data available; evidence is provided in a small number of references; authors report conclusions that vary from one another. Experts have limited experience of the event, and/or there is a moderate level of agreement between experts.
High	There are scarce or no data available; evidence is not provided in references but rather in unpublished reports or based on observations, or personal communication; authors report conclusions that vary considerably between them. Very few experts have experience of the event, and/or there is a very low level of agreement between experts.

**Table 4:** Summary of key findings and assessed likelihoods for risk parameters (L1–L12).

Parameterizations	Key Findings	Likelihood	Uncertainty
L1	76.15% (83/109) checked animal source 61.47% (67/109) quarantined before 84.40% (92/109) vaccinated 94.50% (103/109) daily health checks; critical gap; absence of subclinical carrier detection	Low	Low
L2	77.98% (85/109) sourced roughage externally 84.40% (92/109) cultivated on-farm; notable; uncovered truck beds ~90% (98/109) disinfectant sprays 89.91% (98/109) groundwater use 76.15% (83/109) reported disease history	Low	Moderate
L3	FMD prevalence of pigs 11.11% (2003–2004); farms near manure/markets; lack of comprehensive reservoir control plans; common domestic animals.	Low	Low
L4	92.66% (101/109) awareness of clinical signs 94.50% (103/109) daily observation Significant gap: ~90% (98/109) limited understanding of subclinical carriers 82.57% (90/109) isolated suspected cases 88.99% (97/109) milked infected at the last order 61.47% (67/109) dedicated equipment 93.58% (102/109) never sold milk from clinically infected 83.49% (91/109) ceased deliveries upon detection	High	High
L5	88.99% (97/109) milked infected at the last order 61.47% (67/109) used separate equipment, shared pens 57.80% (63/109) dipped rubber liner, 89.91% (98/109) lifted containers without external cleaning	Low	High
L6	Low PPE usage - Gloves 26.61% (29/109) - Hairnets 42.20% (46/109) - Masks 26.61% (29/109) - Aprons 34.86% (38/109) 70.64% (77/109) not changing clothes before leaving farm 49.54% (54/109) not changing shoes	Moderate	High
L7	75.23% (82/109) brought vehicles near milking pen, inconsistent use of dipping ponds 49.54% (54/109) not regularly cleaning vehicles, no disinfectant use for interiors	Low	High
L8	Wheel-dipping ponds are present Milk delivery personnel risk; duration of wheel-dipping pond contact rarely monitored; no systematic inspection of equipment	Low	Moderate
L9	Staff-personnel interaction risk; inadequate PPE usage (infrequent gloves/aprons); shared equipment as fomites	Moderate	High
L10	65.14% (71/109) washed containers on-farm 77.98% (85/109) returned directly to the farm; on-farm washing has been prevalent since 2020 (COVID-19 protocols)	Low	High
L11	Milk containers delivered directly by drivers (various exposure settings); duration of wheel-dipping pond contact often not monitored; inadequate personal hygiene (not changing clothes or shoes); no disinfection for fomites such as mobile phones	Low	High
L12	88.07% (96/109) engaged in close-contact activities upon return 65.14% (71/109) washed containers near animal pens	High	High

**Table 5:** Summary of the assessed occurrence likelihood for foot-and-mouth disease virus introduction and transmission to dairy farms via raw milk transportation, derived using a descriptive scale and combination matrix based on conditional pathway parameters [32].

Parameterizations	Risk Level	Combined Risk	
**Likelihood of entry**			
Introduction of FMDV into milk			
L1: Presence of FMD-infected animals	Low	Low *	Low *
L2: Contaminated feed and water	Low		
L3: Domestic reservoir transmission	Low		
Contamination during the milking process			
L4: Milk from infected animals mixed with bulk	High	Low *	
L5: Contaminated milking equipment	Low		
L6: Contaminated milker during milking process	Moderate		
L7: Contaminated vehicles during the milking process	Low		
**Likelihood of exposure**			
Contamination during transportation and loading			
L8: Inadequate biosecurity at milk collection center	Low	Low *	Low *
L9: Contaminated personnel and equipment	Moderate		
L10: Washing milk containers at the same site	Low		
Transmission to dairy farms			
L11: Inadequate biosecurity at dairy farms	Low	Low *	
L12: Indirect contact with dairy cattle	High		
**Likelihood of the occurrence of hazard**			
Likelihood of entry	Low	Low *	
Likelihood of exposure	Low		

* Combination matrix adopted.

**Table 6:** Overall risk estimation of foot-and-mouth disease virus outbreaks via raw milk transportation, utilizing a descriptive scale and combination matrix based on unconditional pathway parameters [41].

Parameterizations	Risk Level	Risk Estimation
Likelihood of hazard occurrence	Low	Moderate *
Consequences assessment	High	

* Combination matrix adopted.

## Data Availability

All data are contained within this paper.

## Abbreviations

The following abbreviations are used in this manuscript:

FMD	Foot-and-mouth disease
FMDV	Foot-and-mouth disease virus
PPE	Personal protective equipment
WOAH	The World Organization for Animal Health
